# Levels of antibodies to SARS-CoV-2 at key time points during the COVID-19 pandemic in China

**DOI:** 10.3389/fpubh.2023.1271917

**Published:** 2023-11-22

**Authors:** Liu Lina, Liu Hui

**Affiliations:** Department of Laboratory and Quarantine, Dalian Medical University, Dalian, China

**Keywords:** COVID-19, SARS-CoV-2, antibody, vaccination, public health

## 1 Introduction

Coronavirus disease 2019 (COVID-19), caused by severe acute respiratory syndrome coronavirus 2 (SARS-CoV-2), is a worldwide public health problem that has occurred in recent years. SARS-CoV-2 is found mainly in the respiratory secretions of patients infected with COVID-19. These infected patients are also the main source of transmission, which occurs primarily through the respiratory tract (1). When the virus enters the trachea, the mucosal cells that make up the tracheal surface secrete mucus to encapsulate the virus, which is then propelled by cilia toward the larynx or nose for expulsion. However, COVID-19 is characterized by a dry cough rather than expectoration, suggesting that the virus has crossed the trachea and traveled through the bronchi to the alveoli. The virus enters the alveoli, infects the alveolar cells, and begins to multiply. Immune cells, e.g., macrophages, kill the infected lung cells and engulf the debris, and any remaining debris that is not engulfed needs to be expelled by coughing (2, 3). At the same time, specific immunity can be triggered to produce antibodies and other immune substances, resulting in most people being cured. However, a small proportion of patients experience disease exacerbation, even resulting in death (4, 5).

From a population perspective, the population was not immune to SARS-CoV-2 before 2019 because SARS-CoV-2 is a novel virus. Pneumonia of unknown origin (later named COVID-19) was detected on December 08, 2019 in Wuhan, China (6). As of January 30, 2020, there were 12,050 cases of COVID-19 (confirmed) and 259 deaths in China, with a mortality rate of 2.1% (7).

After January 20, 2020, mainland China adopted the highest level of prevention and control measures, as well as a “zero-COVID” policy, including comprehensive screening of infection sources (asymptomatic infected persons), strict quarantine measures, closure of schools and places where people congregate, wearing of masks, and disinfection of public places. After April 2020, the outbreak of COVID-19 was largely contained, with only a few disseminated cases remaining (7).

COVID-19 vaccines were successfully developed and widely used since 2021, and the vaccination rate (inactivated vaccine) in mainland China exceeded 90% by the middle of 2021 (8). However, mainland China-mainland continued with the highest level of prevention and control measures/“zero-COVID” until the end of 2022. The peak incidence of COVID-19 occurred in late 2022 and early 2023, ending with a peak at end of January 2023, because mainland China no longer adhered to the highest level of prevention and control measures/“zero-COVID”.

Throughout the course of the COVID-19 epidemic, mainland China adopted a variety of preventive and control measures, including non-pharmacological control (administrative interventions) and vaccination. Thus, mainland China is an excellent model to observe the course of the epidemic and evaluate the various preventive and control measures. This article provides SARS-CoV-2 antibody data at major time points during the COVID-19 epidemic in China to provide a scientific basis to study the prevalence of COVID-19 and evaluate various preventive and control measures.

## 2 Materials and methods

Antibody data from the beginning of 2020 were obtained from published literature (9–11). Data from the first half of 2022 and the first half of 2023 were obtained from measured antibody levels. The antibody test samples (serum) were obtained hospitalized patients in The Second Hospital of Dalian Medical University. All patients were free of COVID-19 infection or other infection within 1 week before examination and all had received more than one COVID-19 vaccination. In the 2022 group, the average age of the patients was 58.9 ± 19.1 years old, and there were 517 men and 314 women. In the 2023 group, the average age was 58.9 ± 17.2 years old, and there were 897 men and 960 women. The levels of total antibody to SARS-CoV-2 were tested using a chemiluminescence-based method, and a cutoff value >1.00 was considered positive.

Non-normally distributed data were reported as the median and interquartile range. The significance of differences was determined by Kruskal–Wallis H tests using SPSS software. A value of *P* <0.05 (bilateral) was considered statistical significance.

## 3 Results and discussion

The beginning of 2020 data showed that the population antibody positivity rate was 3.2–6.9% (3.9 were suggested due to relatively large sample size). SARS-CoV-2 was a new virus and the population was not immune; therefore, these data suggest that around 3.9% of the population was infected with COVID-19.

**Figure 1:**
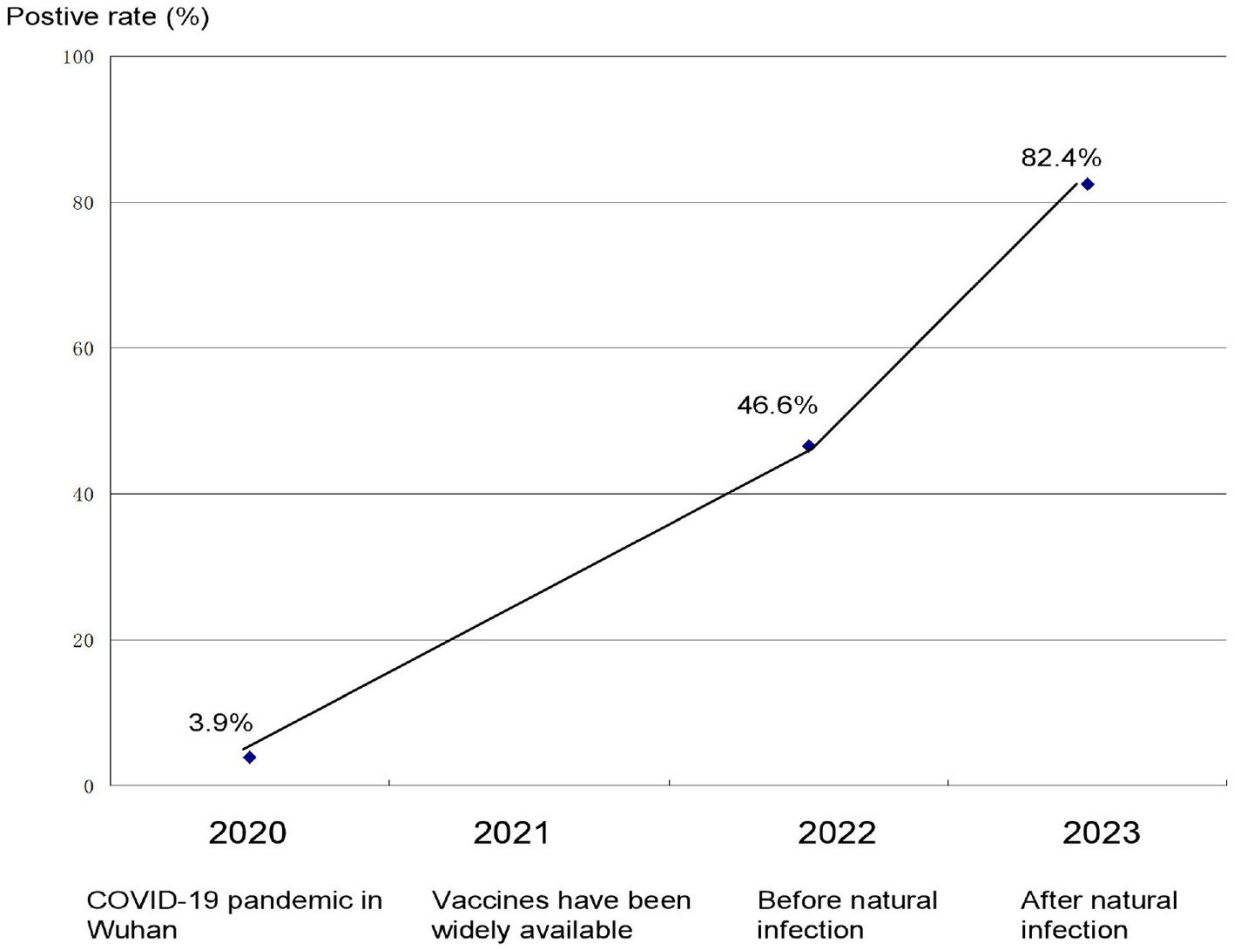
Positive rate of antibody to SARS-CoV-2 at key time points during the COVID-19 pandemic in China.

After the successful development of the vaccine by the end of 2020, the vaccination rate in mainland China exceeded 90% in the end of 2021 (8). Our test data shows that the antibody positive rate of a mainland China (Dalian) population after vaccination was 46.6% in the first half of 2022, as shown in Figure 1. Compared with the pre-vaccination period, in which the antibody positivity rate in mainland China was about 3.9%, while mainland China adopted strict control measures and monitoring positive cases (usually daily testing for key populations; if found positive, immediate population-wide testing); therefore, this result indicated that the antibody positivity rate in the first half of 2022 comes mainly from vaccination rather than from natural infection.

At the end of 2022, the Chinese government adopted normalized prevention and control and relaxed the strict control measures. As a result, the peak incidence of COVID-19 occurred in early 2023. Our data showed that the antibody positivity rate in mainland China (Dalian) was 82.4% in the first half of 2023 after the Chinese government adopted normalized control measures (Figure 1). Obviously, natural infection played an important role in the increase in antibody levels.

We also performed quantitative antibody tests on the antibody-positive population. We found that the level of antibodies to SARS-CoV-2 in early 2023 was significantly higher than that in the January 2022 (derived from the vaccine), as shown in Table 1, indicating that the antibodies detected in early 2023 could mainly derived from natural infection.

**Table 1:** Quantitative antibody to SARS-CoV-2 on the antibody-positive population.

**Times**	**Quartile**			***Z-*value**	***P-*value**
	**25th**	**50th**	**75th**		
First half of 2022	4.520	10.210	25.260	13.785	< 0.001
First half of 2023	7.773	68.660	152.970		

Despite the many variants of SARS-CoV-2, neutralizing antibodies were effective against most of them, and no new serotypes have been identified (12–14). The total antibodies were measured in present study, thus the variants could have little effect on the rate of antibody to SARS-CoV-2 in the population.

The immune imprinting effect is important. Because most countries in the world did not use the Zero-COVID-19 policy as the Chinese government did, the previous study used clinical cohorts whose immune histories are too complex to trace. Thus, Chinese cohort is very “clean” for the study of the immune imprint effect. The quantitative results of the positive antibodies to SARS-CoV-2 had also been provided in present study, which could be used as a reference for the study of the immune imprint effect.

The limitation of this paper is that a population antibody positivity rate of 82.4% does not completely exclude vaccination as the source. However, inactivated vaccines are generally considered to be less effective (15) and show limited interference with antibodies produced by natural infection; thus, the data in this paper remain of great value.

## Data availability statement

The original contributions presented in the study are included in the article/supplementary material, further inquiries can be directed to the corresponding author.

## Ethics statement

Ethical approval was not required for the study involving humans in accordance with the local legislation and institutional requirements. Written informed consent to participate in this study was not required from the participants or the participants' legal guardians/next of kin in accordance with the national legislation and the institutional requirements.

## Author contributions

LL: Data curation, Formal analysis, Investigation, Methodology, Writing—original draft. LH: Conceptualization, Formal analysis, Writing—original draft, Writing—review & editing.
